# Genetic Diversity of *Aeromonas* spp. Isolates from the Paediatric Population in Latvia Based on Multilocus Sequence Typing

**DOI:** 10.3390/children13010111

**Published:** 2026-01-12

**Authors:** Irina Grave, Aleksandra Rudzate, Edvins Miklasevics, Dace Gardovska

**Affiliations:** 1 Faculty of Medicine, Riga Stradins University, LV-1007 Riga, Latvia; aleksandra.rudzate@rsu.lv (A.R.); dace.gardovska@rsu.lv (D.G.); 2 Children Clinical University Hospital, Bernu Kliniska Universitates Slimnica, LV-1004 Riga, Latvia

**Keywords:** *Aeromonas*, gastroenteritis, paediatric, multilocus sequence typing (MLST), genetic diversity

## Abstract

**Purpose**: *Aeromonas* species are emerging pathogens associated with a range of human infections, particularly gastroenteritis in the paediatric population. However, data on their molecular characteristics in Latvia and the wider Baltic region remain limited. This study aimed to describe the distribution and genetic diversity of *Aeromonas* isolates from paediatric patients in Latvia using multilocus sequence typing (MLST). **Methods**: Stool samples were collected from children aged 0–18 years who presented with gastroenteritis at the Children’s Clinical University Hospital between 2020 and 2021. A subset of 30 non-duplicate *Aeromonas* isolates was randomly selected for molecular characterisation and analysed by MLST. Sequence type data were deposited in the PubMLST.org database. **Results**: MLST analysis revealed substantial genetic diversity, with 25 novel sequence types (STs) being identified and three isolates matching previously reported STs. Two novel STs were detected in duplicate, with an epidemiological link being confirmed in one case. The local distribution of *Aeromonas* species and STs differed from global PubMLST datasets, suggesting a distinct local population of *Aeromonas* strains in Latvian paediatric patients. **Conclusions**: This study provides the first MLST-based description of paediatric *Aeromonas* isolates in Latvia and establishes a regional baseline for future comparative and epidemiological research. Although limited by a small sample size, the findings contribute to the molecular characterisation of *Aeromonas* spp. in the Baltic region and support the value of continued surveillance rather than direct public-health inference at this stage.

## 1. Introduction

*Aeromonas* spp. are regarded as ubiquitous in aquatic environments, with isolates recovered from rivers, lakes, ponds, estuarine seawater, drinking water, groundwater, and wastewater, including multiple stages of sewage treatment. Members of this genus tolerate a wide range of temperatures, conductivities, pH values and turbidity levels. In aquatic ecosystems, some species also occur as digestive tract symbionts of fish. In addition, *Aeromonas* spp. have been detected in soil and in various food products, including seafood, dairy, meat, poultry, and vegetables [1,2,3,4,5]. *Aeromonas* species are important pathogens in animals, particularly aquatic species, where they cause a range of systemic and localised infections. They are also recognised as emerging human pathogens associated with gastroenteritis, skin and soft tissue infections, and, less frequently, peritonitis, necrotising fasciitis, osteomyelitis, bacteraemia and pneumonia, particularly in immunocompromised patients [4,6,7,8].

Multilocus sequence typing (MLST) can be employed for most bacterial species and a key advantage of MLST over alternative molecular typing methods is the portability of sequence data across laboratories. MLST characterises bacterial isolates by sequencing internal fragments, typically ~500 base pairs in length, from a defined set of housekeeping genes, usually across seven distinct loci. Each unique sequence at a locus is assigned an arbitrary allele number, and the combination of alleles across all loci defines an allelic profile, which is designated as a specific sequence type (ST). This approach enables the creation of expanding, species-specific global databases, hosted on web-based platforms to facilitate the exchange of molecular typing data and support global epidemiological research [2,9,10,11,12,13,14]. The MLST scheme for *Aeromonas* was developed in 2010 based on data from Martino et al. [2] and includes six housekeeping genes: *gyrB*, *groL*, *gltA*, *metG*, *ppsA*, and *recA*. This scheme is freely accessible for research purposes.

Over the past decade, interest in the clinical significance of infections caused by *Aeromonas* species has increased. Available studies indicate that most clinical isolates belong to four species, with *A. caviae*, *A. veronii* and *A. dhakensis* being the most commonly identified, followed by *A. hydrophila*. Occasional reports also describe clinical isolates of newly recognised species *A. trota*, *A. eucrenophila*, *A. jandaei* and *A. media*. However, variation in species distribution has been reported between studies [1,3,5,6,7,15,16].

The role of *Aeromonas* species in gastroenteritis remains debated. However, these bacteria are recognised as significant pathogens in paediatric care [17,18]. In our previous study, a notable association between *Aeromonas* and diarrhoea was observed, supporting evidence from other publications on their role in paediatric infections [19]. Nevertheless, data from European research remain limited. Accurate identification of *Aeromonas* species is essential to investigate potential associations between infection frequency and the pathogenic mechanisms related to transmission routes.

The aim of this study was to apply MLST for the molecular characterisation and epidemiological investigation of clinical *Aeromonas* isolates from paediatric patients with gastroenteritis in Latvia. Particular attention was given to the broader European context, where data on *Aeromonas* infections remain limited. This study also contributes to the global MLST database and improves understanding of the clinical significance and transmission dynamics of *Aeromonas* spp. in human infections.

## 2. Materials and Methods

### 2.1. Sample Collection

Stool samples were collected at the Children’s Clinical University Hospital between 2020 and 2021. The target population included patients aged 0–18 years with a preliminary diagnosis of gastroenteritis, corresponding to the International Classification of Diseases, 10th Revision (ICD-10) codes A04 (Other bacterial intestinal infections), A09.0 (Other and unspecified gastroenteritis and colitis of infectious origin), and A09.9 (Gastroenteritis and colitis of unspecified origin). Exclusion criteria included repeated hospitalisation for a known infectious gastroenteritis aetiology and patients who had received antibiotic therapy prior to admission. Immunocompromised patients were tested for the full bacterial range and were not excluded solely on the basis of prior antimicrobial exposure. In our previous study, 50 *Aeromonas*-positive stool isolates were identified within this cohort [19]. From this collection, a subset of 30 non-duplicate isolates representing the detected species and age group was randomly selected for pilot molecular characterisation using MLST, with the aim of establishing a local baseline for future expanded genomic surveillance.

### 2.2. Isolation and Identification of Aeromonas spp.

Bacterial isolation was performed using CIN agar plates (Biolife Italiana, Monza, Italy), prepared according to the manufacturer’s instructions. Plates were incubated under aerobic conditions at 28 ± 1 °C for 48 h [13]. In parallel, the same samples were plated onto CIN agar without selective supplements and incubated at 35 ± 1 °C for 24 h. Oxidase testing was performed using Becton Dickinson Oxidase Reagent Droppers. Colonies testing positive were selected for further identification with the Bruker MALDI-TOF Biotyper (Bruker Daltonics, Massachusetts ASV, Bremen, Germany).

The comparison of *Aeromonas* isolation methods, species distribution, antimicrobial susceptibility, presence of virulence-associated genes, as well as their impact on disease course was described in detail in our previous study [19].

### 2.3. Nucleotide Sequencing of Gene Fragments

Total chromosomal DNA from *Aeromonas* spp. was extracted using the Analytik Jena innuPREP Bacteria DNA kit (AJ Innuscreen GmbH, Berlin, Germany, following the manufacturer’s instructions. Nucleotide sequences of internal fragments from the following housekeeping genes (corresponding protein products are provided in parentheses) were obtained from the *Aeromonas* MLST database [20]: *gyrB* (DNA gyrase β subunit), *groL* (chaperonin GroEL), *gltA* (citrate synthase I), *metG* (methionyl-tRNA synthetase), *ppsA* (phosphoenolpyruvate synthase), and *recA* (recombinase A). Primer designs were obtained from previous studies [2], and sequences are provided in Supplementary Table S1.

PCR amplification was performed using primers synthesised by Metabion International. Reactions were carried out in a final volume of 50 µL, containing 5 µL of 10× PCR buffer, 2 µL of 2.5 mM dNTP mix, 6 µL of MgCl_2_, 1 µL of forward primer, 1 µL of reverse primer (working stock 10 µM; final concentration 0.2 µM each), 1 µL of DNA template (50 ng/µL), 0.2 µL of Taq polymerase (5 U/µL), and 33.8 µL of nuclease-free water (ddH_2_O). Thermal cycling was performed with an initial denaturation step at 95 °C for 5 min, followed by 35 cycles of denaturation at 95 °C for 30 s and annealing at 55–62 °C for 30 s, according to the primer melting temperature specified by the manufacturer. Extension was carried out at 72 °C for 1 min, with a final extension at 72 °C for 7 min. Amplification products were pre-cooled to 15 °C and stored at −20 °C until further analysis. PCR products were subsequently visualised by agarose gel electrophoresis and submitted to Sanger sequencing. Sequencing results were compared with the curated, open-access PubMLST database collection, and the allelic profiles, along with the corresponding MLST sequence types and isolate information, were submitted to the database [20].

## 3. Results

Thirty *Aeromonas* spp. isolates from paediatric patients were analysed in this study. The median patient age was 5 years (range: 0.75–17 years), with equal sex distribution (15 males and 15 females). A total of 25 novel sequence types (STs) were identified by comparison of concatenated gene sequences (*gyrB*, *groL*, *gltA*, *metG*, *ppsA*, *recA*) using the PubMLST database (Table 1). Two novel MLST sequence types, ST3403 and ST3438, were identified in duplicate; however, a potential epidemiological link (patients from the same family) was established in only one case. In addition, three isolates matched previously reported STs, specifically ST737 and ST181, with the two ST181 isolates being identical. PubMLST accession identifiers and corresponding isolate metadata are provided in Supplementary Table S2.

The distribution of *Aeromonas* species in Latvia (*n* = 30) differed from the global dataset (*n* = 4184) (Table 2). However, these findings should be interpreted with caution due to the relatively small Latvian sample size. *A. caviae* was the predominant species locally, accounting for 56.7% of isolates, compared with 27.92% globally. Similarly, *A. hydrophila* comprised 23.3% of Latvian isolates versus 10.52% worldwide. In contrast, *A. veronii*, the most common species globally (28.87%), represented only 16.7% of the local isolates. Notably, *A. eucrenophila* was identified exclusively in the Latvian dataset, while several other species associated with human infections, including *A. dhakensis*, *A. jandaei*, and *A. media* [1,3,5,6,7,15,16], were absent locally but reported in the global collection.

## 4. Discussion

This study provides the first multilocus sequence typing (MLST) data for *Aeromonas* spp. from Latvia, contributing 30 isolates to the global MLST database, and enhancing the molecular epidemiological representation of the European Union (EU). Previous research in Latvia has focused primarily on the clinical prevalence of *Aeromonas* infections in paediatric patients with gastroenteritis [19]. Together, these findings improve understanding of the geographic distribution, genetic diversity, and epidemiological patterns of *Aeromonas* spp. in Latvia within the wider European region.

When placed in a global context, the contribution of Latvian isolates becomes clearer. To date, a total of 4184 bacterial isolates have been reported in the PubMLST database, originating from 36 distinct countries [20]. Most isolates were reported from Asia, with China (21.44%) and India (17.02%) contributing the largest proportions. Notably, 15.99% of isolates lacked specific geographic origin information, indicating gaps in reporting. Within the European Union (EU), isolate distribution varied between member states. Spain accounted for 7.22% of isolates (302 records), followed by Italy with 3.73% (156 records), Latvia and Poland with 0.72% (30 records) each, and Denmark with 0.57%. Portugal, France, Norway, Germany, and Belgium each represented less than 1% of the isolates. No data were available from other EU countries. It is important to note that the isolates represent a range of sources, including those human infections, environmental or animal origins, and isolates with unreported or unknown sources.

Currently, the global *Aeromonas* MLST database includes 3628 sequence types (STs) [20], reflecting the potentially high genetic diversity of this genus. The 25 distinct STs identified in our study, representing approximately 0.69% of all known types, further demonstrate considerable genetic variation, even within a small regional sample. One of these, ST737 (*A. caviae*), was previously isolated from the stool of a healthy contact and reported by Shuang Meng at the National Institute for Communicable Disease Control and Prevention, China CDC. Another, ST181 (*A. caviae*), was isolated in Spain from the stool of a patient with diarrhoea. Notably, ST181 was identified in two of our isolates, but no epidemiological link could be established between the corresponding patients, suggesting that these strains could be widely distributed and may be independently acquired in distinct geographic regions.

This diversity, alongside the absence of clear epidemiological links between cases, highlights the complex transmission dynamics and potential for localised genetic differentiation of *Aeromonas* populations. In a small number of isolates, MALDI-TOF and MLST yielded different species assignments. In these cases, identical allelic profiles across all MLST loci indicated that the isolates belonged to the same genetic lineage. The discrepant MALDI-TOF identifications are consistent with previously reported limitations of MALDI-TOF in resolving closely related *Aeromonas* species and underline the value of complementary sequence-based approaches for species assignment. At the same time, limited data from the Baltic region and other EU countries, together with uneven geographic representation, continue to constrain understanding of infection sources and transmission pathways. These gaps highlight the need for enhanced surveillance and standardised reporting across the EU. In addition, the substantial proportion of isolates without specified origins points to ongoing challenges in data completeness. The observed differences in *Aeromonas* species distribution between our local dataset and the global collection suggests geographic variation in species prevalence and emphasises the importance of regional surveillance for understanding local epidemiology and informing potential public health strategies.

It is important to note that clinical samples for this investigation were collected during the COVID-19 pandemic, a period characterised by social distancing and other public health interventions [19]. These measures may have limited opportunities for bacterial transmission within the population, particularly in settings such as childcare facilities, and may therefore have influenced the observed genetic diversity of MLST types by reducing strain circulation and limiting the formation of new transmission chains. In addition, the small sample size (n = 30), randomly selected for this pilot study, constrains the generalisability of the findings. Future studies with larger cohorts and conducted in post-pandemic settings will be essential to more comprehensively elucidate the epidemiological dynamics and diversity of *Aeromonas* spp. infections in Latvia and the wider European region.

## Figures and Tables

**Table 1:** Allelic profiles and corresponding MLST sequence types. Novel sequence types (STs) identified in this study are highlighted in blue, while white cells indicate previously described STs in the PubMLST database.

Sample Nr.	*Aeromonas* Species	gyrB	groL	gltA	metG	ppsA	recA	MLST
001	*A. hydrophila*	1	978	254	842	405	437	2809
002	*A. hydrophila*	797	482	969	518	1297	73	3354
003	*A. caviae*/*A. hydrophila* *	278	610	96	159	639	294	3403
004	*A. hydrophila*	1006	948	960	159	671	1033	3417
008	*A. caviae*	86	94	89	88	92	510	737
009	*A. caviae*	152	12	96	159	667	402	3418
010	*A. caviae*	282	102	96	96	97	516	3419
011	*A. caviae*	341	335	96	159	851	366	3420
012	*A. caviae*	529	510	96	159	249	159	3421
013	*A. hydrophila*	962	652	254	842	405	437	3422
014	*A. veronii*	1087	940	940	785	840	432	3423
015	*A. caviae*	152	163	96	159	164	159	181
016	*A. caviae*/*A. hydrophila* *	278	610	96	159	639	294	3403
017	*A. caviae*	282	162	96	159	1111	158	3424
018	*A. caviae*	206	12	96	159	249	173	3425
021	*A. veronii*	552	1044	44	685	345	106	3426
022	*A. caviae*	93	314	96	159	97	173	3427
023	*A. caviae*	152	163	96	159	164	159	181
024	*A. caviae*	152	102	96	96	97	516	3428
029	*A. caviae*	206	162	96	96	587	514	3429
030	*A. caviae*	278	314	200	536	164	173	3430
031	*A. caviae*	278	267	96	96	639	294	3431
032	*A. caviae*	193	611	829	96	174	610	3432
036	*A. eucrenophila*	1098	1041	1170	45	216	171	3433
038	*A. hydrophila*	274	265	375	366	1120	400	3434
041	*A. caviae*	193	12	101	536	164	158	3435
042	*A. hydrophila*	962	652	254	842	405	611	3436
043	*A. veronii*	68	154	311	148	341	330	3437
048	*A. veronii*	340	310	753	755	794	751	3438
049	*A. veronii*	340	310	753	755	794	751	3438

* In isolates 003 and 016, species identification was performed by MALDI-TOF MS. Although they were reported as *A. hydrophila* and *A. caviae,* respectively, both isolates shared identical allelic profiles across all six MLST loci and were assigned the same novel sequence type (ST3403). This strongly suggests that they represent the same strain. The discrepant MALDI-TOF results likely reflect recognised limitations of this method in distinguishing closely related *Aeromonas* species [1,2,11,12].

**Table 2:** Comparative distribution of *Aeromonas* species in Latvian isolates (n = 30) and the global dataset (n = 4184).

*Aeromonas* Species	Number of Isolates from Latvia	Frequency in Latvia (%)	Number of Isolates from Global Dataset	Frequency in Global Dataset (%)
*A. caviae*	17	56.7	1168	27.92
*A. hydrophila*	7	23.3	440	10.52
*A. veronii*	5	16.7	1208	28.87
*A. eucrenophila*	1	3.3	4	0.1
*A. dhakensis*	0	0	251	6
*A. jandaei*	0	0	76	1.82
*A. media*	0	0	52	1.24
*A. sobria*	0	0	51	1.22

## Data Availability

The original contributions presented in this study are included in the article/Supplementary Materials. Further inquiries can be directed to the corresponding authors.

## Supplementary Materials

The following supporting information can be downloaded at https://www.mdpi.com/article/10.3390/children13010111/s1. Table S1: Primers used for MLST analysis [2]; Table S2: PubMLST accession identifiers, sequence type (ST) assignments, and associated metadata for Aeromonas isolates included in this study.
