# Supplementary material for: Genetic Diversity of Aeromonas spp. Isolates from the Paediatric Population in Latvia Based on Multilocus Sequence Typing
Source: Children (Basel). 2026 Jan 12;13(1):111. doi: 10.3390/children13010111 (PMC12840421; doi:10.3390/children13010111)
Supplement: Supplementary file 1 [file children-13-00111-s001.zip › Supplementary Table S1.pdf]

Primer	Sequence (5′-3′)	Gene product	Size of PCR amplicon (bp)
gyrB_F	GGGGTCTACTGCTTCACCAA	DNA gyrase, β subunit	669
gyrB_R	CTTGTCCGGGTTGTACTCGT		
groL_F	CAAGGAAGTTGCTTCCAAGG	Chaperonin GroEL	782
groL_R	CATCGATGATGGTGGTGTTC		
gltA_F	TTCCGTCTGCTCTCCAAGAT	Citrate synthase I	626
gltA_R	TTCATGATGATGCCGGAGTA		
metG_F	TGGCAACTGATCCTCGTACA	Methionyl-tRNA synthetase	657
metG_R	TCTTGTTGGCCATCTCTTCC		
ppsA_F	AGTCCAACGAGTACGCCAAC	Phosphoenolpyruvate synthase	619
ppsA_R	TCGGCCAGATAGAGCCAGGT		
recA_F	AGAACAAACAGAAGGCACTGG	Recombinase A	640
recA_R	AACTTGAGCGCGTTACCAC		

Supplementary Table S1. Primers used for MLST analysis [2].
