# Supplementary material for: Genetic Diversity of Aeromonas spp. Isolates from the Paediatric Population in Latvia Based on Multilocus Sequence Typing
Source: Children (Basel). 2026 Jan 12;13(1):111. doi: 10.3390/children13010111 (PMC12840421; doi:10.3390/children13010111)
Supplement: Supplementary file 1 [file children-13-00111-s001.zip › Supplementary Table S2.pdf]

Supplementary Table S2. PubMLST accession identifiers, sequence type (ST) assignments, and associated metadata for Aeromonas isolates included in this study.

PubMLST isolate ID	Study isolate ID	Country	Year	Species	ST
4104	001	Latvia	2020	*A. hydrophila*	2809
4106	002	Latvia	2020	*A. hydrophila*	3354
4107	003	Latvia	2020	*A. hydrophila*	3403
4108	004	Latvia	2020	*A. hydrophila*	3417
4109	008	Latvia	2020	*A. caviae*	737
4110	009	Latvia	2020	*A. caviae*	3418
4111	010	Latvia	2020	*A. caviae*	3419
4112	011	Latvia	2020	*A. caviae*	3420
4113	012	Latvia	2020	*A. caviae*	3421
4114	013	Latvia	2020	*A. hydrophila*	3422
4115	014	Latvia	2020	*A. veronii*	3423
4116	015	Latvia	2020	*A.caviae*	181
4117	016	Latvia	2020	*A. caviae*	3403
4118	017	Latvia	2020	*A.caviae*	3424
4119	018	Latvia	2020	*A.caviae*	3425
4120	021	Latvia	2020	*A. veronii*	3426
4121	022	Latvia	2020	*A. caviae*	3427
4222	023	Latvia	2020	*A.caviae*	181
4223	024	Latvia	2020	*A.caviae*	3428
4224	029	Latvia	2020	*A.caviae*	3429
4225	030	Latvia	2020	*A.caviae*	3430
4226	031	Latvia	2020	*A.caviae*	3431
4227	032	Latvia	2020	*A.caviae*	3432
4228	036	Latvia	2021	*A. eucrenophila*	3433

PubMLST isolate ID	Study isolate ID	Country	Year	Species	ST
4229	038	Latvia	2021	*A. hydrophila*	3434
4230	041	Latvia	2021	*A.caviae*	3435
4231	042	Latvia	2021	*A. hydrophila*	3436
4232	043	Latvia	2021	*A.veronii*	3437
4233	048	Latvia	2021	*A.veronii*	3438
4234	049	Latvia	2021	*A. veronii*	3438
